# Mother–Child Biobehavioral Synchrony and Its Association With Social Functioning in Autistic School‐Aged Children

**DOI:** 10.1002/aur.70168

**Published:** 2025-12-31

**Authors:** Carly Moser, Chih‐Hsiang Yang, Abigail L. Hogan, Amanda Fairchild, Jane Roberts, Jessica Klusek

**Affiliations:** ^1^ Department of Communication Sciences and Disorders University of South Carolina Columbia South Carolina USA; ^2^ Department of Exercise Science University of South Carolina Columbia South Carolina USA; ^3^ Carolina Autism and Neurodevelopment (CAN) Research Center University of South Carolina Columbia South Carolina USA; ^4^ Department of Psychology University of South Carolina Columbia South Carolina USA

**Keywords:** autism, biobehavioral synchrony, heart activity, parent–child synchrony, respiratory sinus arrhythmia, social functioning

## Abstract

Parent–child biobehavioral synchrony, or the concordance of behavior and physiological indicators between individuals, is theorized to support children's social development; however, this relationship has yet to be investigated in autistic children. This study examined whether moment‐to‐moment physiological synchrony—indexed via respiratory sinus arrhythmia (RSA)—and its interface with global levels of behavioral synchrony was associated with the pragmatic language skills and friendship quality of school‐aged autistic children in 40 mother–child dyads. Mother–child dyads participated in a collaborative task, from which RSA synchrony and behavioral synchrony were assessed. Mothers and their autistic children demonstrated negative RSA synchrony, such that when one partner displayed an increase in RSA, the other partner showed a decrease in RSA. The extent of behavioral synchrony between mothers and their children did not moderate the strength of concordance between mother and child RSA. Negative RSA synchrony was associated with better pragmatic language skills in autistic children from mother–child dyads who displayed high levels of behavioral synchrony. These findings highlight the complexity of dyadic synchrony, suggesting that the coordination of mother–child RSA, in conjunction with behavioral synchrony, may aid in the development of social skills in autistic children that extend beyond the immediate caregiver context. However, larger longitudinal studies are needed to confirm this.

## Lay Summary

This study examined whether mothers and their school‐aged autistic children have consistent heart activity patterns when interacting with each other, which is called physiological synchrony. We also explored whether physiological synchrony was linked to how closely their behaviors aligned, which is called behavioral synchrony. We found that mothers and their autistic children did match their heart activity but in the opposite direction—when one partner had an increase in heart activity, the other partner had a decrease. This is called negative physiological synchrony. In this study, we did not find a link between behavioral synchrony and physiological synchrony. We also explored whether physiological synchrony and behavioral synchrony are related to pragmatic language, or the social use of language, and friendship quality in autistic children. Friendship quality was not related to physiological or behavioral synchrony, but for mothers and children who displayed high levels of behavioral synchrony, more negative physiological synchrony was related to better pragmatic language skills for autistic children.

## 1 Introduction

Interpersonal synchrony, or patterns of mutually regulated and reciprocal interaction, between caregivers and their children is theorized to play a vital role in the development of children's social functioning through repeated exchanges that form a mutually regulated feedback loop supporting social learning (Feldman 2012b; Harrist and Waugh 2002; Sameroff and Fiese 2000). Difficulties with social communication and interaction, including establishing and maintaining social reciprocity, are core features of autism (American Psychiatric Association 2013) that could impact the synchrony of interactions in this population. Indeed, interpersonal synchrony has been shown to be reduced in dyads that include autistic individuals and various interaction partners (e.g., parents, non‐autistic and autistic interaction partners; Carnevali et al. 2024; Georgescu et al. 2020; Zampella et al. 2020, but see Glass and Yuill 2024). While interpersonal synchrony has historically been measured via observable behaviors, research following a biobehavioral synchrony theoretical framework has begun to consider the concordance of physiological indicators, such as autonomic activity, between individuals as being a central aspect of interpersonal synchrony (Feldman 2012a, 2012b; Feldman et al. 2013; Palumbo et al. 2017). Given documented differences in autonomic nervous system function in autistic individuals (found in many studies, though not universally; Arora et al. 2021; Cheng et al. 2020), the study of physiological coordination along with behavioral synchrony (i.e., biobehavioral synchrony) may be instrumental in informing potential explanations for social communication difficulties in autistic individuals. Therefore, the current study examined the presence of mother–child biobehavioral synchrony, indexed by the interplay between physiological and behavioral synchrony, and whether it relates to social functioning in school‐aged autistic children.

Physiological synchrony, or the dyadic coordination of physiological states, is thought to form the foundation for developing more complex social exchanges over time (Feldman 2015) and may serve as a potential biological indicator of social reciprocity (Dunsmore et al. 2019) but this has yet to be systematically investigated in the context of autism. Physiological synchrony can occur when partners are exhibiting physiological changes in the same direction—both increasing or decreasing (i.e., positive physiological synchrony) or when partners are exhibiting physiological changes in opposite directions—one increasing and one decreasing (i.e., negative physiological synchrony). Recent findings in the general population have demonstrated that the adaptive nature of positive and negative physiological synchrony is context‐specific. Positive physiological synchrony is generally assumed to be advantageous; however, it can be maladaptive, occurring in the form of a stress contagion in the context of high family or marital stress, for instance (Levenson and Gottman 1983; Suveg et al. 2016). Likewise, negative physiological synchrony can be adaptive (e.g., a mother who is sensitive to their child's regulatory needs, maintaining a calm demeanor that helps their child regulate in the midst of stress) or maladaptive (e.g., due to high levels of stress, a mother is disengaged when their child needs regulatory support; Creavy et al. 2020; Fuchs et al. 2021; Lunkenheimer et al. 2021; Suveg et al. 2019). The investigation of social functioning outcomes and the inclusion of moderators, such as behavioral synchrony, can aid in further understanding the consequences of the strength and direction of physiological synchrony.

Two preliminary investigations have examined the effect of behavioral synchrony on physiological synchrony in the context of autism (Baker et al. 2015; Saxbe et al. 2017). Baker et al. (2015) examined 28 autistic children aged 4–10 years and their primary caregivers. They measured the coordination of electrodermal activity, an index of the sympathetic branch of the autonomic nervous system, and parent–child affective attunement. Results indicated that stronger and more positive parent–child electrodermal activity synchrony (i.e., electrodermal activity increasing and decreasing in similar patterns in parents and their children) was associated with increased affective attunement (i.e., parents and children displayed balanced exchanges of emotion). Notably, autism traits moderated electrodermal activity synchrony, such that children with fewer autism traits showed stronger parent–child electrodermal synchrony. Another report by Saxbe et al. (2017) examined cortisol synchrony in 40 autistic preschoolers and their parents compared to 40 neurotypical preschoolers and their parents and investigated whether cortisol synchrony was moderated by dyadic behaviors, including behavioral synchrony. The report found that positive mother–child (but not father–child) cortisol synchrony was stronger in neurotypical dyads than in autism dyads. Additionally, dyads with lower levels of parent–child behavioral synchrony showed stronger cortisol synchrony across both groups. These findings suggest that the coordination of cortisol may form a stress contagion where one partner reflects the dysregulation of the other partner. Overall, findings from these studies demonstrate that features related to autism are associated with the presence and strength of physiological synchrony across different biological indicators and that behavioral synchrony can serve as a useful marker of whether and when physiological synchrony is adaptive.

A biological indicator that holds promise in informing physiological synchrony in autism is respiratory sinus arrhythmia (RSA), or the variation in heart rate associated with respiration. The polyvagal theory posits that vagal regulation of the heart, measured by RSA, supports social engagement (Porges 2007). Given the social nature of interpersonal synchrony and evidence of RSA dysregulation in autistic individuals (Cheng et al. 2020), it is important to examine the presence and strength of RSA synchrony in this group. To date, only one study has applied RSA to the investigation of parent–child physiological synchrony in autism (Wang et al. 2021). Wang and colleagues found that while positive RSA synchrony was detected in dyads of parents and their neurotypical children, RSA synchrony was only present in dyads of parents and their autistic children who displayed better interaction quality—a construct closely related to behavioral synchrony. Notably, in the study by Wang et al., RSA was measured every 30 s of the interaction, which could have masked changes in the coordination of physiology that may have been detected using advanced methods to estimate RSA at more frequent intervals (Gates et al. 2015).

Although synchronous exchanges of physiology and behavior between parents and children have been theorized to jointly support children's psychosocial development (Feldman 2012b), existing empirical studies testing this premise remain limited. There is growing recognition that the interaction context shapes the adaptive nature of physiological synchrony (Davis et al. 2018; DePasquale 2020), and behavioral synchrony may be a key contextual factor that facilitates or constrains the extent to which RSA synchrony contributes to developmental outcomes. Behavioral synchrony has consistently been linked to positive social functioning outcomes, including enhanced social skills, peer acceptance, prosocial behavior, and emotional adjustment (Barber et al. 2001; Criss et al. 2003; Lindsey et al. 2008; Pasiak and Menna 2015). Given its role in children's social development, it is possible that the relationship between RSA synchrony and social outcomes is likely modified by whether the quality of the interaction reflects positive, mutually regulated exchanges or disjointed, less behaviorally attuned exchanges.

Understanding the development of social functioning is especially critical for autistic children, considering difficulties with social communication are a core feature of autism. Moreover, social functioning, including the obtainment of mutually reciprocated friendships and pragmatic language skills, can have cascading effects on mental health and objective outcomes (e.g., employment success) in adulthood (Burt et al. 2008; Jones et al. 2015; Masten et al. 2010). The relationship between biobehavioral synchrony and social functioning during the school‐aged years is of particular interest because it is a critical developmental period for the formation of intimate and reciprocal friendships (Laursen and Hartup 2002). Thus, investigating how physiological synchrony interacts with behavioral synchrony in relation to social functioning outcomes in school‐aged autistic children has important implications for providing targeted support.

### 1.1 The Present Study

The present study aims to provide answers to specific gaps in the field of biobehavioral synchrony by quantifying second‐by‐second changes in RSA synchrony among mothers and their school‐aged autistic children, examining the potential moderation of behavioral synchrony, and identifying the role of biobehavioral synchrony on positive social functioning. Specifically, the study investigated the association between autistic school‐aged children and mothers' moment‐to‐moment RSA (RSA synchrony) and whether global levels of behavioral synchrony moderated RSA synchrony. We hypothesized behavioral synchrony would moderate the relationship between child and mother moment‐to‐moment RSA, such that stronger, more positive RSA synchrony would occur in dyads that demonstrate higher levels of behavioral synchrony, but not among dyads with low behavioral synchrony. We also aimed to understand how mother–child RSA synchrony and its interface with behavioral synchrony relate to social functioning (i.e., pragmatic language skills and friendship quality) in autistic school‐aged children. We hypothesized that stronger, more positive RSA synchrony would be associated with better social functioning in autistic children, particularly when levels of mother–child behavioral synchrony were high.

## 2 Method

### 2.1 Participants

Participants were 40 autistic children (*M*_age_ = 8 years) and their mothers (*M*_age_ = 40 years), recruited from the southeastern region of the United States, including North Carolina, South Carolina, and Georgia. All parents were eligible but only mothers enrolled in the study. Four additional dyads were initially recruited for the study but were excluded from the sample due to issues with physiological data collection (i.e., more than 5% of the signal contained artifacts for either the child or mother participant). To avoid medication confounds for cardiac data, dyads were excluded if either the mother or the child was taking cardioactive medications (e.g., beta‐blockers), tricyclic antidepressants, or clozapine. Other psychotropic medications (e.g., SSRIs) were not excluded, given they have little influence on heart activity (Alvares et al. 2016). Inclusion criteria were as follows: (1) children were 7–10 years of age, (2) English was the primary language spoken in the home, (3) children could communicate using 2–3‐word phrases, per caregiver report, which was necessary for the pragmatic language measure, and (3) parents reported their child had received an autism diagnosis in the past, with community diagnoses confirmed with the Autism Diagnostic Observation Schedule, Second Edition (ADOS‐2; Lord et al. 2012) as part of the research study. Families were recruited via social media and word of mouth. Additionally, flyers were posted in local pediatricians' offices and circulated through the Carolina Autism and Neurodevelopment Research Registry. Characteristics of the sample are presented in Table 1.

**TABLE 1:** Descriptive statistics of the sample and main study variables.

Variable	*M* (SD) or *n*	% or range
Sample characteristics		
Child sex at birth		
Male	32	80%
Female	8	20%
Child age	8.81 (1.22)	7.04–10.97
Child race		
Asian	1	2%
Black	10	25%
White	25	63%
More than one race	4	10%
Child nonverbal IQ^a^	99.67 (22.90)	40.00–147.00
Child autism symptom severity^b^	6.60 (1.72)	4.00–10.00
Mother age	40.27 (6.25)	23.27–53.48
Mother education		
No bachelor's degree	14	35%
Bachelor's degree or higher	23	58%
Not reported	3	7%
Main study variables		
Child RSA	5.59 (0.88)	3.62–7.17
Mother RSA	4.69 (1.08)	1.29–6.38
Dyadic behavioral synchrony	3.46 (0.96)	1.50–5:00
Child pragmatic language skills	23.95 (7.08)	12.00–38.00
Child friendship quality	2.36 (1.04)	1.00–5.00

^a^ Measured using the matrices subtest of the KBIT‐2.

^b^ Measured using the calibrated severity score of the ADOS‐2.

### 2.2 Procedures

Informed consent was obtained from mothers, and children verbally assented prior to participation in the study. All procedures were approved by the University of South Carolina's Institutional Review Board. Dyads were tested in a laboratory at the University of South Carolina (*n* = 26) or in a quiet area of their home (*n* = 14). First, heart rate monitors were placed on mothers and children. Children then completed the Kaufman Brief Intelligence Test, Second Edition (KBIT‐2; Kaufman and Kaufman 2004) and the ADOS‐2. Mothers filled out questionnaires focused on their child's behavior, development, and social participation during this time. Finally, mothers and their children engaged in a 4‐min interaction task, where the dyad was instructed to copy drawings (a house and a tree) using an Etch‐A‐Sketch (Stevenson‐Hinde and Shouldice 1995). Each partner was instructed to use one knob to foster communication and elicit goal‐oriented behaviors from the dyad. The Etch‐A‐Sketch task has been used in several studies investigating dyadic synchrony in school‐aged children, including those with autism (Suveg et al. 2016; Wang et al. 2021, Han et al. 2019).

### 2.3 Measures

#### 2.3.1 Behavioral Synchrony

Behavioral synchrony was indexed from the videotaped interaction of the Etch‐A‐Sketch task using the dyadic reciprocity code of the Coding Interactive Behavior (CIB) global rating system (Feldman 1998). The dyadic reciprocity code assesses the degree to which the dyad participates equally in the joint activity, responds to their partner's cues, and engages in well‐coordinated exchanges. The dyadic reciprocity code is rated on a 5‐point Likert scale ranging from a “1” (indicating the behavior is rarely seen) to a “5” (indicating the behavior is seen often). The CIB has been used in studies of neurotypical and autistic children (Levy et al. 2017; Pratt et al. 2017; Saxbe et al. 2017) and shows good psychometric properties (Feldman 2012c). Prior to the study, one rater established reliability with the authors of the CIB. A second rater was trained by achieving greater than 80% agreement with the established rater on three consecutive practice videos. All videos were coded by a primary rater (graduate‐level research assistant) and second coding was conducted on a randomly selected 20% of the sample (*n* = 8) to establish inter‐rater reliability. The intra‐class correlation (ICC) for the dyadic reciprocity codes was 0.89 in the sample (indicating good reliability).

#### 2.3.2 Moment‐To‐Moment RSA Estimation

Physiological data were collected during the Etch‐A‐Sketch task with the Actiheart‐5 monitor (CamNTech)—a small, lightweight, wireless monitor that attaches to the chest using two electrodes and captures an ECG signal sampled at 1024 Hz. The ECG signal was processed through CardioEdit Plus software (CardioEdit Plus Software 2022) to correct for artifacts. Once the ECG signals were cleaned, RSAseconds—a program freely available online (https://gateslab.web.unc.edu/programs/rsaseconds/)—was used to apply a time‐series transformation that provides 1‐s estimates of RSA across the interaction (Gates et al. 2015; Hansson and Jönsson 2006). Briefly, RSAseconds employs a peak matched multiple window technique that applies a series of overlapping 32‐s windows (shifted by 1 s) across the IBI signal. Each segment combines four tapered windows that emphasize the center of the window to produce more reliable RSA estimates; thus, the time series begins 16 s into the task and ends 16 s before end of the Etch‐A‐Sketch task. Within each window, RSA was computed using a short‐time Fourier transformation with appropriate respiratory frequency bands (0.12–0.40 Hz for mothers; 0.12–1.00 Hz for school‐aged children). The absolute values are squared to obtain power estimates, followed by a natural log transformation, yielding a time series of second‐by‐second RSA estimates. For more details on this technique, see Gates et al. (2015).

#### 2.3.3 Assessment of Social Functioning

##### 2.3.3.1 Pragmatic Language Skills

Pragmatic language skills were measured using the Pragmatic Rating Scale‐School Age (PRS‐SA; Landa 2011), which shows strong interrater reliability and concurrent validity (Dillon et al. 2021). The PRS‐SA rates pragmatic behaviors during a semi‐naturalistic social interaction. The ADOS‐2, which lasts approximately 45 min, was used as the social interaction from which to rate pragmatic language abilities, as recommended by the developer of the PRS‐SA. The PRS‐SA consists of 32 items that index behaviors related to pragmatic language, including theory of mind, discourse management, speech, language, and nonverbal communicative behaviors. Each item is rated and scored on a scale from 0 to 2, with a 0 representing “typical behavior” and a 2 representing “atypical behavior.” The total score, defined as the sum of all item scores, was used in the analysis with higher scores representing more pragmatic language violations. Raters were trained to reliability by achieving greater than 80% agreement with an established rater on four out of five consecutive files. A primary rater (graduate‐level research assistant) coded the PRS‐SA for all participants. Inter‐rater reliability was conducted on a randomly selected 20% (*n* = 8) of the sample (ICC = 0.78; good reliability).

### 2.4 Friendship Quality

The quality of children's friendships was measured using a modified version of the GO4KIDDS survey (Perry and Weiss 2008), a parent‐report measure that has been used to capture social participation, including the formation and maintenance of friendships, in autistic children (Taheri et al. 2016). The survey probes for the frequency of participation in several types of activities, the child's number of friends, and the quality of their friendships, using a 5‐point Likert scale. The quality of friendship rating was used in the analysis with a 1 indicating “very poor” and a 5 indicating “excellent.”

### 2.5 Data Analysis

To address the first research question, examining child and mother RSA synchrony, a general linear mixed effects model with a first‐order autoregressive structure, which accounts for the non‐independence of cardiac data, was conducted using the nlme package in R (Pinheiro et al. 2022; R Core Team 2022). Mother RSA was modeled as the dependent variable and child RSA was modeled as the independent variable.^1^ Two variables were created from each child's RSA values to account for between‐ and within‐person effects, following Bolger and Laurenceau (2013). Between‐person effects (average RSA) were modeled using grand‐mean centering, where each child's average RSA across the Etch‐A‐Sketch task was centered around the sample mean and was included in the model as a potential covariate. Within‐person effects (moment‐to‐moment RSA) were modeled using person‐mean centering by subtracting their average RSA across the task from each one‐second segment, with a positive value indicating an increase from their average and a negative value indicating a decrease from their average. Moment‐to‐moment RSA (within‐person effect) was the independent variable of interest. To determine the best‐fitting model, a model‐building approach was used to compare random effects structures and the inclusion of potential covariates. Potential covariates included: child average RSA (between‐person effect), to account for individual differences in average RSA and potential biologically related concordance in RSA between mother–child dyads; time from task onset (within‐person effect) to account for changes in mother RSA across the task (Bolger and Laurenceau 2013); child race (between‐person effect), captured dichotomously as White vs. non‐White due to the limited sample sizes in several racial categories, to account for potential cultural influences on interpersonal synchrony (Deater‐Deckard et al. 2004; Lindsey et al. 2008); and visit setting (between‐person effect; lab vs. home). All models that were considered are reported in supplemental materials. The best‐fitting model was a mixed effects model that included a random effect for child moment‐to‐moment RSA. Of the covariates tested, only time improved model fit and was therefore retained in the final model.

To address the second research question, examining the moderating effect of behavioral synchrony, an interaction term between behavioral synchrony (grand‐mean centered) and child moment‐to‐moment RSA was added to the model. A significant interaction between behavioral synchrony and moment‐to‐moment RSA would indicate that the influence of momentary changes in child RSA on mother RSA varied by the level of synchrony in their behavioral interactions.

To test the third research question regarding the relationship between biobehavioral synchrony and social functioning, an estimate of RSA synchrony was created by conducting a random slope and intercept mixed model. Child moment‐to‐moment RSA was the independent variable, and mother moment‐to‐moment RSA was the dependent variable. Time across the interaction was included in the model as a covariate. The predicted estimates of the random slope for each mother–child dyad were extracted as a measure of within‐dyad RSA synchrony. Then, two models were fit with RSA synchrony (within‐dyad random slope estimate) and behavioral synchrony as predictors of pragmatic language skills and friendship quality. The interaction between RSA synchrony and behavioral synchrony was added to the model. To align with the type and distribution of each outcome variable, a general linear model was used to predict pragmatic language skills, and an ordinal logistic regression was used to predict friendship quality. Child race and visit setting were probed as potential covariates but did not improve model fit, so they were not retained in the final models. All independent variables were grand‐mean centered. Visual inspection of the residual plots for the general linear models showed linearity, homoscedasticity, and normality, and Cook's distance values fell below 0.1, suggesting no influential observations. To assess the proportional odds assumption for the ordinal logistic regression, we conducted a Brant test, which indicated that the proportional odds assumption was not violated (*p* = 0.300). Additionally, there was no evidence of multicollinearity for any model (VIFs = 1). Partial eta squared ($\eta_{p}^{2}$) effect sizes were computed using the effectsize package in R (Ben‐Shachar et al. 2020) for the general linear model, with effects of 0.01, 0.06, and 0.14 representing small, medium, and large effects, respectively (Cohen 1988).

## 3 Results

### 3.1 Child Moment‐To‐Moment RSA and Its Association With Mother Moment‐To‐Moment RSA

The model estimates of the mixed effects model regressing mother RSA on child RSA are presented in Table 2. Child moment‐to‐moment RSA was a significant predictor of mother moment‐to‐moment RSA, such that mothers and their autistic children demonstrated negative physiological synchrony (i.e., coordinated responses but with members of the dyad moving in opposite directions). In other words, at any given moment during the interaction, a decrease in the child's RSA (i.e., RSA suppression) was associated with increases in the mothers' RSA (i.e., RSA augmentation), and vice versa.

**TABLE 2:** Associations between child and mother moment‐to‐moment RSA.

	Estimate	SE	*p*
Intercept	4.69	0.17	< 0.001
Time	−0.05	0.00	< 0.001
Child moment‐to‐moment RSA	−0.25	0.11	0.021

Random effects	Estimate	Intercept‐slope correlation	
Intercept	1.05		
Child moment‐to‐moment RSA	0.69	0.18	
Residual	0.72		

*Note:* Fixed effects estimates represent unstandardized beta coefficients. Random effects estimates reflect standard deviations for the intercept (i.e., variation in mother RSA across individuals), slope (i.e., variation in the effect of child RSA on mother RSA across individuals), and residual (i.e., variation in mother RSA not explained by the model). The intercept–slope correlation indicates how mother‘s average RSA level relates to the strength of the association between mother and child RSA.

Abbreviation: SE, standard error.

### 3.2 Behavioral Synchrony Moderating Moment‐To‐Moment and Average RSA Synchrony

The model estimates of the mixed effects model testing the moderating effect of behavioral synchrony on the association between mother and child RSA are presented in Table 3. The degree of dyadic behavioral synchrony did not moderate the relationship between mother and child moment‐to‐moment RSA.

**TABLE 3:** Associations between child and mother moment‐to‐moment RSA and the moderating effect of behavioral synchrony.

	Estimate	SE	*p*
Intercept	4.69	0.17	< 0.001
Time	−0.05	0.00	< 0.001
Behavioral synchrony	0.11	0.18	0.541
Child moment‐to‐moment RSA	−0.25	0.11	0.023
Behavioral synchrony × child moment‐to‐moment RSA	−0.02	0.12	0.834

Random effects	Estimate	Intercept‐slope correlation	
Intercept	1.06		
Child moment‐to‐moment RSA	0.70	0.18	
Residual	0.72		

*Note:* Fixed effects estimates represent unstandardized beta coefficients. Random effects estimates reflect standard deviations for the intercept (i.e., variation in mother RSA across individuals), slope (i.e., variation in the effect of child RSA on mother RSA across individuals), and residual (i.e., variation in mother RSA not explained by the model). The intercept–slope correlation indicates how mother‘s average RSA level relates to the strength of the association between mother and child RSA.

Abbreviation: SE, standard error.

### 3.3 Mother–Child Biobehavioral Synchrony as a Predictor of the Social Functioning of Autistic Children

**FIGURE 1:**
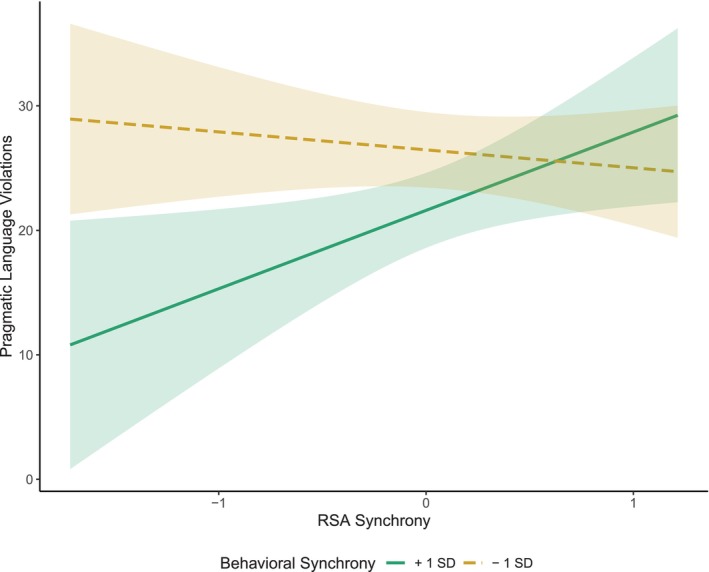
Behavioral synchrony moderating the relationship between RSA synchrony and children's pragmatic language skills. *Note:* High behavioral synchrony is shown in green (1 SD above the mean). Low behavioral synchrony is shown in yellow (1 SD below the mean). More positive values of RSA synchrony indicate mother and child's RSA are coordinated and responding in the same direction. More negative values of RSA synchrony indicate mother and child's RSA are coordinated but responding in the opposite direction.

Model estimates and effect sizes of the general linear model testing the main effects and interaction between behavioral and RSA synchrony on pragmatic language skills are presented in Table 4. Before testing the interaction, neither the main effect of RSA nor the main effect of behavioral synchrony was significant. After the interaction term was entered into the model, the main effect of RSA synchrony was not significant but behavioral synchrony accounted for significant variance in the model, indicating that higher levels of behavioral synchrony were associated with fewer pragmatic language violations. A significant interaction between RSA synchrony and behavioral synchrony was detected, with a medium‐to‐large effect size, indicating that the influence of RSA synchrony on the pragmatic language skills of autistic children varied according to the level of behavioral synchrony exhibited by the dyad (see Figure 1). Follow‐up simple slopes analyses indicated a significant association between RSA synchrony and pragmatic language for children in dyads with high behavioral synchrony (1 SD above the mean; *ß =* 6.30, *p* = 0.024), such that children in dyads with more negative mother–child RSA synchrony (RSA responses are coordinated between mother and child but in opposite directions) demonstrated fewer pragmatic language violations and children in dyads with more positive mother–child RSA synchrony demonstrated more pragmatic language violations. Among the dyads that displayed low behavioral synchrony (1 SD below the mean), RSA synchrony was not associated with pragmatic language violations (*ß =* −1.44, *p* = 0.457).

**TABLE 4:** Estimates from general linear models predicting children's pragmatic language skills.

Predictor	Model 1				Model 2			
	Estimate	SE	*p*	$\eta_{p}^{2}$	Estimate	SE	*p*	$\eta_{p}^{2}$
Intercept	23.95	1.10	< 0.001		24.04	1.04	< 0.001	
RSA synchrony	1.23	1.63	0.456	0.02	2.43	1.63	0.144	0.06
Behavioral synchrony	−2.02	1.15	0.088	0.08	−2.53	1.11	0.029	0.13
Behavioral synchrony × RSA synchrony	*—*	—	*—*	*—*	4.02	1.72	0.025	0.13

*Note:* Estimates are unstandardized coefficients.

Abbreviation: SE, standard error.

The ordinal logistic regression testing behavioral synchrony, RSA synchrony, and their interaction as predictors of friendship quality showed no significant main effect of RSA synchrony, no main effect of behavioral synchrony, and no interaction between RSA synchrony and behavioral synchrony. All model estimates are presented in Table 5.

**TABLE 5:** Estimates from ordinal logistic regressions predicting children's friendship quality.

Predictor	Model 1		Model 2			
	Estimate	SE	*p*	Estimate	SE	*p*
RSA synchrony	0.08	0.43	0.851	0.23	0.43	0.595
Behavioral synchrony	−0.01	0.32	0.983	−0.12	0.34	0.718
Behavioral synchrony × RSA synchrony	*—*	*—*	*—*	0.71	0.48	0.134

*Note:* Estimates are unstandardized coefficients.

Abbreviation: SE, standard error.

## 4 Discussion

The present study offers novel insights into the interpersonal synchrony between mothers and their autistic children, providing important implications for the potential role of biobehavioral synchrony in the development of social functioning. During a collaborative task, mothers and their autistic children demonstrated discordant moment‐to‐moment RSA synchrony, such that, at any given moment, when one partner displayed RSA augmentation, the other partner displayed RSA suppression. Behavioral synchrony did not moderate this relationship, which challenges the assumption that physiological and behavioral synchrony are dependent on one another, at least in the context of autism. However, we did find that behavioral synchrony moderated the relationship between mother–child RSA synchrony and autistic children's pragmatic language skills. These findings highlight the complexity of dyadic synchrony, and though future work is needed to replicate these findings with a larger sample using a longitudinal framework, our results suggest that the coordination of mother–child RSA may work in conjunction with behavioral synchrony to provide a foundation for autistic children's social functioning skills that transfer beyond the caregiver context.

Contrary to our hypothesis, findings demonstrate evidence of *negative* moment‐to‐moment RSA synchrony among mothers and their autistic children. Our results differ from those of Wang et al. (2021), who used the same interaction context (the Etch‐A‐Sketch task) in a group of similarly aged autistic children and found positive RSA synchrony but only in dyads with higher interaction quality. These differences may be due to cultural interaction differences (the Wang et al. sample was Chinese) or, at least in part, to our higher temporal resolution of RSA sampling (captured every second as opposed to every 30 s; Gates et al. 2015). Rapid modulation of arousal within small windows of time is important to account for, as it is more temporally aligned with behavioral responses to the social environment (Keller et al. 1999). In fact, our findings do align with another study that employed the same Etch‐A‐Sketch task with 71 mothers and their preschool children, measuring physiological synchrony every 10 s (Suveg et al. 2016). Suveg et al. found that in high‐risk dyads (e.g., less educational attainment, economic disadvantage, and high maternal stress), negative physiological synchrony was associated with more positive behavioral synchrony and greater self‐regulation skills. Thus, this type of collaborative and goal‐oriented interaction may elicit negative physiological synchrony as an adaptive co‐regulatory reaction for certain populations (e.g., high‐risk families; families with autistic children), which can be captured when using smaller time intervals to index physiological concordance.

In the current study, behavioral synchrony did not moderate concordance between mother and child RSA, which is inconsistent with the findings of Baker et al. (2015) who found behavioral synchrony, as indexed via affective attunement, was associated with EDA synchrony. These divergent findings may be due to differences in physiological methods (EDA indexes sympathetic activity while RSA reflects parasympathetic activity) and behavioral modalities. Using the same measure of behavioral synchrony from the CIB, Saxbe et al. (2017) did not find evidence of an interaction between dyadic reciprocity and cortisol synchrony for mother–child dyads, which aligns with our current study. However, evidence of this association was found for father‐child dyads, which suggests parent–child behavioral synchrony—defined as a mutual adaptation to a partner's behavior with well‐coordinated exchanges—may differentially interact with physiological synchrony based on parent gender or other aspects of parenting behavior. While current theories underscore the importance of the interplay between physiological synchrony and behavioral synchrony (Feldman 2006, 2007), our study adds to the growing body of research that has not detected a relationship between physiological and behavioral synchrony across certain contexts and populations (Armstrong‐Carter et al. 2021; Motsan et al. 2021; Woltering et al. 2015).

Although behavioral synchrony was not directly related to RSA synchrony, these constructs worked interdependently to predict pragmatic language skills, highlighting the importance of continuing to examine these modalities of synchrony together. Specifically, RSA synchrony was associated with pragmatic language skills for dyads who displayed high levels of behavioral synchrony, such that more negative RSA synchrony was associated with better pragmatic language skills, and more positive RSA synchrony was related to worse pragmatic language skills. While this differs from earlier research in which it was assumed that stronger, more positive physiological synchrony was adaptive, these results corroborate more recent literature finding that negative RSA synchrony can also be advantageous in specific contexts (Creavy et al. 2020; Lunkenheimer et al. 2021; Suveg et al. 2016). Given that behaviorally synchronous dyads displaying negative RSA synchrony had stronger pragmatic language skills, it is probable these mothers and children were sensitive to one another's behavior and regulatory states in a way that aids in the child's social learning. In other words, these results may be taken to indicate that children in more collaborative and sensitive dyads can translate their learned social interaction skills with their mother to other social contexts, which aligns with prominent developmental theories, such as the transactional model of development and attachment theory (Bowlby 1969, 1978; Sameroff 2009; Sameroff and Fiese 2000).

Autistic children with higher levels of behavioral synchrony but more positive RSA synchrony tended to have worse pragmatic language skills. While these dyads may be sensitive to one another's behavioral and regulatory states in the interaction, this sensitivity may translate into a stress contagion, where dysregulated stress responses may be taken on by the other partner. As such, children may not be able to use these parent–child interactions as an effective foundation for social communication across other contexts. A stress‐contagion explanation has been proposed in prior work examining mother–child biobehavioral synchrony, including in autistic samples (e.g., Saxbe et al. 2017; Suveg et al. 2016; Waters et al. 2014), but should be met with caution in the context of this study, as we did not explicitly account for the emotional valence of interaction partners or systematically manipulate the stress levels of the dyad.

Interestingly, RSA synchrony was not associated with enhanced or diminished pragmatic language skills in dyads with less behavioral synchrony. In dyads of children and mothers who demonstrated low levels of behavioral synchrony, there was little sense of reciprocity, and partners typically displayed independent actions during the interaction task. Therefore, it may be that RSA synchrony is only linked to social functioning if there is adequate behavioral engagement and reciprocity. Low behavioral synchrony could reflect reduced social attention, a feature documented among autistic individuals (Chita‐Tegmark 2016), which may be important for facilitating stronger physiological synchrony that translates to more positive social outcomes (Behrens et al. 2020). On the other hand, disengagement could stem from different interaction styles within the dyad. According to the double empathy problem, defined as a breakdown in mutual understanding (Milton et al. 2022), a mismatch in communication styles and social preferences may lead to a less synchronous interaction (Glass and Yuill 2024). It is possible that with a different interaction partner with similar interaction styles, greater reciprocity could emerge and facilitate a stronger link between RSA synchrony and social functioning. Future studies should account for autistic traits across both partners to better understand the effect the interaction partners may have on biobehavioral synchrony and social outcomes.

Unexpectedly, no relationship was found between biobehavioral synchrony and friendship quality. While interpersonal synchrony is theoretically linked with the ability to form and maintain friendships (Feldman 2012b), the effects of dyadic synchrony on social skills (e.g., pragmatic language skills) have been more consistently documented (Criss et al. 2003; Harrist et al. 1994; Lindsey et al. 1997; Mize and Pettit 1997). Thus, friendship quality may be too distally related to mother–child dyadic synchrony. Our findings may also be limited by our measure of friendship quality, which was indexed via parent report. Self‐reported perception of friendship or observational studies of friendship quality may be better suited to accurately represent peer relationships in autistic youth.

The present study has several strengths. First, extant research has rarely focused on the interplay between multiple modalities of mother–child synchrony and their relationship to social development (Davis et al. 2018). Our study confirmed the importance of including multiple modalities of synchrony, as they demonstrate an interactive effect on developmental outcomes. Additionally, the use of advanced methods to index second‐by‐second changes in RSA (Gates et al. 2015) allowed us to capture moment‐to‐moment RSA synchrony. Despite these strengths, the present study has several limitations and offers potential avenues for future research. Due to the cross‐sectional nature of the study, we are not able to make causal inferences about the relationship between biobehavioral synchrony and social functioning skills. Additionally, we were not adequately powered to systematically investigate potential cultural differences due to our limited sample size. Due to sample limitations, fathers were not included in the present study, yet they play a distinct and crucial role in autistic children's social development (e.g., Saxbe et al. 2017); the examination of triadic (e.g., mother–father‐child) family interactions is also an important area of future study. Although physiological synchrony was observed, it is unclear whether synchrony reflects true concurrent coordination, if mothers are responding to their child's physiological state, children are responding to their mother's physiological state, or a combination of these possibilities. Future studies with a larger sample that can employ a lagged approach may be better suited to address this question. Additionally, this study only examined a single measure of physiological synchrony within a specific task; future studies should compare multiple measures of physiological synchrony across contexts to determine under which conditions the current findings may hold. Finally, the two measures of synchrony—behavior and RSA—were measured on different timescales. In general, differences in the time metrics of measurements make it challenging to compare estimates across modalities, and there is currently no consensus on optimal synchronization across systems or how to best integrate multilevel synchrony data that operate at different timescales. However, differences in timescales of synchrony across modalities do have implications for how findings may be interpreted and generalized. Micro‐level analyses of both behavioral and physiological synchrony may better capture the temporal relationship between the two modalities and could explain the lack of association in our study. On the other hand, macro‐level measures of behavior can provide more ecologically valid insight into how the overall rhythm of an interaction across behavioral modalities relates to or interacts with moment‐to‐moment RSA synchrony. This study contributes to the ongoing effort to better understand under which conditions different modalities of synchrony are and are not related to one another, which will help to advance a more unified biobehavioral synchrony construct.

In conclusion, the present study offers novel insights into interpersonal synchrony between mothers and autistic children, demonstrating evidence of negative RSA synchrony. These data also contribute to a better understanding of the role of mother–child biobehavioral synchrony on the social functioning of autistic children and underscore the need to include several modalities of synchrony to accurately interpret their influence on development.

## Funding

This work was supported by the University of South Carolina Maternal Child Health Catalyst Program, sponsored by the U.S. Department of Health and Human Services Health Resources and Services Administration (1‐T1CMC35361‐01‐00) and a SPARC Graduate Research Grant from the Office of the Vice President for Research at the University of South Carolina.

## Conflicts of Interest

The authors declare no conflicts of interest.

## Supporting information


**Data S1:** Supporting Information.

## Data Availability

The data that support the findings of this study are available on request from the corresponding author. The data are not publicly available due to privacy or ethical restrictions.

## Endnotes

^1^
We modeled coordination between mother and child physiology with child RSA as the independent variable and mother RSA as the dependent variable, because at this developmental stage, interactions are increasingly child‐led, with mothers tending to respond to the behavioral and physiological states of their children, adapting to their needs (Birk et al. 2022; Harrist and Waugh 2002). Secondly, given autonomic differences observed in autistic individuals, they may be more likely to be the drivers of the physiological attunement throughout the interaction. We probed a separate model with mother moment‐to‐moment RSA as a predictor of child RSA, which was not significant (*p* = 0.490), suggesting mothers were more likely to be influenced by their child's physiology.
