# Supplementary material for: Mother–Child Biobehavioral Synchrony and Its Association With Social Functioning in Autistic School‐Aged Children
Source: Autism Res. 2025 Dec 31;19(2):e70168. doi: 10.1002/aur.70168 (PMC12948655; doi:10.1002/aur.70168)
Supplement: Supplementary file 1 — Data S1: Supporting Information. [file AUR-19-0-s001.docx]

Supplemental Table 1. Results from models considered during the model-building process for RQ1: associations between mother and child RSA.

|  | Model 1 | Model 2 | Model 3 | Model 4  (Final Model) | Model 5 | Model 6 | Model 7 |
| --- | --- | --- | --- | --- | --- | --- | --- |
| *Fixed Effects* |  |  |  |  |  |  |  |
| Intercept | 4.69 (0.17)*** | 4.69 (0.17)*** | 4.69 (0.17)*** | 4.69 (0.17)*** | 4.69 (0.16)*** | 4.78 (0.27)*** | 4.85 (0.28)*** |
| Child moment-to-moment RSA | -- | -0.16 (0.01)*** | -0.26 (0.11)* | -0.25  (0.11)* | -0.25 (0.11)* | -0.25 (0.11)* | -0.25 (0.11)* |
| *Covariates* |  |  |  |  |  |  |  |
| Time | -- | -- | -- | -0.05 (0.00)*** | -0.05 (0.00)*** | -0.05 (0.00)*** | -0.05 (0.00)*** |
| Child average RSA | -- | -- | -- | -- | -0.21 (0.19) | -- | -- |
| White (vs not White) | -- | -- | -- | -- | -- | -0.15 (0.34) | -- |
| In-lab visit (vs home visit) | -- | -- | -- | -- | -- | -- | -0.25 (0.34) |
| *Random Effects* |  |  |  |  |  |  |  |
| Intercept | 1.06 | 1.06 | 1.04 | 1.03 | 1.02 | 1.03 | 1.03 |
| Child moment-to-moment RSA | -- | -- | 0.68 | 0.68 | 0.68 | 0.68 | 0.68 |
| Residual | 0.68 | 0.68 | 0.72 | 0.72 | 0.72 | 0.72 | 0.72 |
| *Model Fit* |  |  |  |  |  |  |  |
| AIC | 4869.41 | 4696.86 | 3097.77 | 2901.41 | 2902.15 | 2903.22 | 2902.88 |
| BIC | 4898.07 | 4732.68 | 3147.92 | 2958.72 | 2966.63 | 2967.70 | 2967.36 |
| *Likelihood Ratio Test* |  |  |  |  |  |  |  |
|  |  | vs Model 1 | vs Model 2 | vs Model 3 | vs Model 4 | vs Model 4 | vs Model 4 |
|  | -- | 174.55*** | 1603.09*** | 198.36*** | 1.26 | 0.19 | 0.53 |

Note. Maximum likelihood estimation was used to compare models, though Restricted Maximum Likelihood was used for final models reported in the Results. AIC=Akaike’s Information Criterion; BIC=Bayesian Information Criterion. Fixed effects estimates represent unstandardized beta coefficients with standard error in the parentheses. Random effects estimates reflect standard deviations for the intercept, slope, and residual.

^*^*p*<..05; ***p*<.01; ****p*<.001
